# Paxillin regulates vascular endothelial growth factor A-induced *in vitro* angiogenesis of human umbilical vein endothelial cells

**DOI:** 10.3892/mmr.2014.2961

**Published:** 2014-11-17

**Authors:** WAN-JU YANG, YAN-NING YANG, JIN CAO, ZI-HUI MAN, YING LI, YI-QIAO XING

**Affiliations:** 1 Eye Center, Renmin Hospital of Wuhan University, Wuhan, Hubei 430060, P.R. China; 2 Department of Ophthalmology, The Central Hospital of Wuhan, Tongji Medical College, Huazhong University of Science and Technology, Wuhan, Hubei 430014, P.R. China

**Keywords:** paxillin, vascular endothelial growth factor, endothelium, angiogenesis

## Abstract

The purpose of the present study was to investigate the role of paxillin in the vascular endothelial growth factor A (VEGF-A)-induced adhesion, proliferation, migration and capillary formation of endothelial cells (ECs) *in vitro*. Human umbilical vein ECs (HUVECs) were used to evaluate these four processes *in vitro*. The HUVECs were either mock-transfected (control), transfected with scramble small interference RNA (siRNA) or transfected with siRNA specifically targeting paxillin. VEGF-A (20 ng/ml) was used to stimulate angiogenesis. The VEGF-A treatment significantly increased the adhesion, proliferation, migration and tube formation of the HUVECs in the control and scramble siRNA groups, whereas the siRNA-mediated knockdown of paxillin inhibited these VEGF-A-induced effects. Paxillin is essential for VEGF-A-mediated angiogenesis in ECs and its inhibition may be a potential target for antiangiogenic therapies.

## Introduction

Corneal avascularity, the absence of blood vessels in the cornea, is required for corneal transparency and the maintenance of vision. Corneal neovascularization, which is induced by a wide range of inflammatory, infectious and traumatic disorders, can lead to visual impairment and even blindness (1,2). It has been reported that 4.14% of ophthalmic patients in the USA have corneal neovascularization (3). Corneal angiogenesis is a common histopathological feature of corneal diseases, leading to corneal transplantation (4). Although several medical and surgical options are currently available for the management of corneal neovascularization, treatment remains challenging and problematic (5).

Under normal physiological conditions, corneal avascularity is maintained by a balance between high levels of antiangiogenic factors and low levels of angiogenic factors (1,5). The presence of neovascularization indicates the activation of several angiogenic factors promoting angiogenesis, which is likely to be associated with a downregulation of antiangiogenic factors (6,7). Several angiogenic molecules have been identified, including vascular endothelial growth factors (VEGFs), fibroblast growth factors and matrix metalloproteinases (MMPs) (1). VEGFs have been identified as being important in the angiogenic process, which involves the proliferation, migration and differentiation of endothelial cells (ECs) and the degradation of the surrounding extracellular matrix (ECM) (1,8,9).

VEGF-A, also termed VEGF 165, is a member of the VEGF family that is important in angiogenesis. VEGF-A binds to two tyrosine kinase receptors, VEGFR1 and VEGFR2. The latter receptor, VEGFR2, is the direct signal transducer for angiogenesis. By comparison, VEGFR-1 has weaker kinase activity and negatively modulates VEGFR-2-mediated angiogenesis (10). The binding of VEGF-A to VEGFR2 triggers several signaling pathways, including the activation of phosphoinositide 3-kinase (PI3K) and Akt, the recruitment of protein lipase C (PLC) and subsequent activation of the mitogen-activated protein kinase (MAPK) cascade through protein kinase C (PKC) and the activation of focal adhesion kinase (FAK) and its substrate paxillin (9,11). In addition, the activation of VEGFR2 eventually leads to the differentiation, proliferation, migration and capillary formation of ECs (9,11,12).

Paxillin is a signal transduction adaptor protein, which is associated with focal adhesion and is one of the major substrates of FAK, a nonreceptor protein tyrosine kinase (13). Paxillin can be phosphorylated on Tyr 31 (PY31) and Tyr 118 (PY118) by FAK or Src kinases (14). VEGF-A has been reported to recruit FAK, which phosphorylates paxillin in ECs (15,16). This phosphorylation promotes the formation of the paxillin-Crk-Dock180 molecular complex (17,18), regulates the activity of Rho guanine triphosphatase (19,20) and activates the Rac and extracellular signal-regulated kinase (ERK) signaling pathways (17,21,22), which leads to increases in cell migration and adhesion (14). However, the role of paxillin in the VEGF-A-induced proliferation, migration, adhesion and capillary formation of ECs remains to be elucidated.

The purpose of the present study was to investigate the role of paxillin in the VEGF-A-induced proliferation, migration, adhesion and capillary formation of human umbilical vein ECs (HUVECs) through the small interfering (si)RNA-based knockdown of paxillin.

## Materials and methods

The present study was performed in accordance with the Declaration of Helsinki and the use of human cells/tissues was approved by the Medical Ethical Committee of Wuhan University (Wuhan, China). HUVECs were isolated from two female patients (24 and 30 years old). Written informed consent was obtained prior to cell/tissue collection

### HUVEC cell culture

The HUVECs, which were isolated from human umbilical cord veins, as previously reported by Jaffe *et al* (23), were cryopreserved following primary culture and stored at the Department of Ophthalmology of Wuhan University. The HUVECs were seeded into poly-L-lysine-coated flasks and maintained in endothelial complete medium supplemented with 5% fetal bovine serum, 1% penicillin/streptomycin and 1% EC growth supplement (ScienCell Research Laboratories, San Diego, CA, USA). The cells were maintained at 37°C in a humidified incubator under 5% CO_2_, with the medium replaced every 2–3 days until the cells reached confluency. The cells were harvested with 0.05% trypsin-ethylene glycol tetraacetic acid solution (Wuhan Boster Bio Engineering Co., Ltd., Wuhan, China) and were further cultured in the poly-L-lysine-coated flasks for use in the subsequent experiments, which used cells starting at passage five when they exhibited a cobblestone appearance.

### Von Willebrand factor immunofluorescence staining

The HUVECs were grown on glass coverslips in sterile six-well plates until they reached confluency. The cells were rinsed with phosphate-buffered saline (PBS; Wuhan Boster Bio Engineering Co., Ltd.) three times and fixed with 4% paraformaldehyde for 30 min at room temperature (RT). The cells were permeabilized with 0.1% Triton X-100 for 15 min and then incubated in a 3% H_2_O_2_/ethanol solution to inhibit the endogenous peroxidase. The cells were then washed with PBS three times and were incubated with the primary antibody, polyclonal rabbit anti-human von Willebrand factor (1:100; Wuhan Boster Bio-Engineering Co., Ltd., Wuhan China), at 4°C overnight. PBS without primary antibodies was used as a negative control. After 24 h, the primary antibody was removed by washing the cells with PBS and the immunoreactivity was detected by incubating the cells with the fluorescein isothiocyanate-coupled secondary antibody, goat anti-rabbit immunoglobulin (Ig)G (1:10; Wuhan Boster Bio-Engineering Co., Ltd.), at RT for 45 min. Cell nuclei were counter-stained with 4,6-diamino-2-phenylindole (Wuhan Boster Bio Engineering Co., Ltd.). The coverslips were then washed with PBS, the cells were examined with a fluorescence microscope (Olympus, Tokyo, Japan) and images were captured with a DP70 digital camera (Olympus).

### Immunoprecipitation

The HUVECs were grown to confluence and stimulated with 20 ng/ml VEGF-A (Cell Signaling Technology, Inc., Beverly, MA, USA) at 37°C for 0, 20, 40 and 60 min. The cells were then washed with ice-cold PBS and solubilized on ice with lysis buffer containing 150 mM NaCl, 10 mM Tris-HCl, (pH 7.5) and 1% Triton X-100 supplemented with a cocktail of phosphatase and proteinase inhibitors containing 1 mM vanadate, 10 mg/ml leupeptin, 10 mg/ml aprotinin, 1 mM phenylmethylsulfonyl fluoride and 0.36 mM phenanthroline. The lysates were centrifuged at 10,000 xg for 15 min at 4°C and the supernatants were incubated with polyclonal mouse anti-human paxillin (Abcam, Cambridge, MA, USA), anti-mouse IgG and protein A-agarose at 4°C overnight. The immunoprecipitates were then collected by centrifugation and the agarose pellet was suspended in 2X SDS-PAGE buffer. The expression of total paxillin and phosphorylated paxillin was determined by western blot analysis.

### Knockdown of paxillin in the HUVECs

The HUVECs were seeded into six-well plates at a density of 5×10^6^ cells/ml. The cells were maintained overnight at 37°C in a humidified incubator supplemented with 5% CO_2_ followed by transfection with a duplex of oligonucleotides targeting paxillin mRNA using Lipofectamine^TM^ 2000 (Invitrogen Life Technologies, Carlsbad, CA, USA). All the siRNA constructs were obtained from Guangzhou RiboBio Co., Ltd. (Guangzhou, China). The following three pairs of paxillin siRNAs were used: siRNA1 forward, 5′GCUGGAACUGAACGCUGUAdTdT3′ and reverse, 5′UACAGCGUUCAGUUCCAGCdTdT3′ (target sequence: GCTGGAACTGAACGCTGTA); siRNA2 forward, 5′GUGUGGAGCCUUCUUUGGUdTdT3′ and reverse, 5′ACCAAAGAAGGCUCCACACTdTd3′ (target sequence: GTGTGGAGCCTTCTTTGGT); and siRNA3 forward, 5′GCAGCAACCUUUCUGAACUdTdT3′ and reverse, 5′AGUUCAGAAAGGUUGCUGCTdTd3′ (target sequence: GCAGCAACCTTTCTGAACT). A scramble siRNA construct was used as a negative control. The efficiency of the siRNA-mediated paxillin-knockdown was examined by reverse transcription quantitative polymerase chain reaction (RT-qPCR) and western blot analysis.

### RT-qPCR

Total RNA was isolated from the HUVECs using TRIzol reagent (Invitrogen Life Technologies) according to the manufacturer’s instructions. The RNA was reverse-transcribed into complementary DNA using a Plexor™ qPCR System (Promega Corporation, Madison, WI, USA). The RT-qPCR was performed with a final volume of 20 μl containing 2 μl cDNA, 0.5 μl of each primer and 10 μl SYBR green. The primers used for the amplification of paxillin are shown in Table I. The β-actin gene was used as a control housekeeping gene. The cycling parameters were as follows: 95°C for 20 sec, followed by 40 cycles of 95°C for 10 sec, 60°C for 20 sec and 70°C for 1 sec, with a final extension at 65°C for 15 sec. Melting curve analyses were performed to verify the amplification specificity. The mRNA expression ΔCt values of paxillin from each sample were calculated by normalizing against the internal control β-actin and the relative expression of paxillin was calculated using the 2^−ΔΔCT^ method (24).

### Western blot analysis

The HUVECs were homogenized on ice in lysis buffer after 48 h siRNA treatment. The proteins were resolved by SDS-PAGE and transferred onto polyvinylidene difluoride membranes by electroblotting. The membranes were inhibited with 10% non-fat dry milk in Tris-buffered saline with Tween-20 (pH 8.0) and incubated with the polyclonal primary antibody rabbit anti-human paxillin (1:1,000; Epitomics, Burlingame, CA, USA). For immunoprecipitation, a primary antibody against phosphorylated paxillin at Y118 and Y31 (polyclonal rabbit anti-human p-paxillin; 1:1,000; Epitomics) was also used. The membranes were then incubated with the antibodies at 4°C overnight. β-actin was used as a loading control. The membranes were then incubated with a polyclonal horseradish peroxidase-linked goat anti-rabbit secondary antibody (1:5,000; KPL, Gaithersburg, MD) at RT for 2 h. The bands were visualized using a chemiluminescence detection system with Novex^®^ ECL chemiluminescent substrate reagent kit (Pierce, Rockford, IL, USA)

### Cell adhesion assay

Following siRNA and VEGF-A treatment, the HUVECs in the experimental and control groups were harvested and counted. Equal quantities of cells (1×10^5^ cells/ml) were seeded into 96-well plates coated with Matrigel (Wuhan Boster Bio Engineering Co., Ltd.) and cultured for 2 h at 37°C in a humidified incubator with 5% CO_2_. Following incubation, the cells were washed twice with Hank’s solution to remove the non-adherent cells. Images of the adherent cells were captured followed by further incubation with 20 μl MTT (5 mg/ml, Wuhan Boster Bio Engineering Co., Ltd.) for 4 h at RT. The optical density of the plates was measured at a wavelength of 490 nm using a microplate reader (Model 550; Bio-Rad, Tokyo, Japan).

### Cell proliferation assay

The cell proliferation was evaluated using an 5-ethynyl-2′-deoxyuridine (EdU) Cell Proliferation assay kit (Molecular Probes, Invitrogen Life Technologies) according to the manufacturer’s instructions. Briefly, the HUVECs, grown in multiwell plates, were incubated in 100 μl endothelial complete medium containing 50 μM EdU (Wuhan Boster Bio Engineering Co., Ltd.) for 2 h. The cells were washed twice with PBS and fixed with 4% paraformaldehyde in PBS for 30 min at RT. The cells were then washed with glycine (2 mg/ml) for 5 min in a shaker and permeabilized with 0.5% Triton X-100 for 10 min. The cells were again washed twice with PBS and incubated in 1X Apollo^®^ reaction buffer, containing 100 mM Tris-HCl (pH 8.5), 1 mM CuSO_4_, 100 μm Apollo 550 fluorescent azide and 100 mM ascorbic acid, for 30 min at RT, protected from light. The cells were then permeabilized three times with 0.5% Triton X-100, followed by 1–2 washes with 100 μl methanol. The cells were subsequently stained with Hoechst 33342 for 30 min at RT, protected from light, and washed with PBS.

Images were captured and analyzed using a BD Pathway 855 High Content Bioimager (BD Biosciences, San Jose, CA, USA). A total of 16 random fields were selected in each well. The number of EdU-positive cells and Hoechst-stained cells were calculated and the EdU-positive cells were expressed as the ratio of the total cell number. These ratios were normalized to the control ratios.

### Cell cycle analysis by flow cytometry

The HUVECs were harvested, washed twice in ice-cold PBS and centrifuged at 160 × g for 5 min. They were then resuspended in chilled ethanol (250 ml/l) and incubated at 4°C overnight. Subsequently, the cells were washed with PBS, stained with propidium iodide for 30 min and analyzed by flow cytometry. Data were acquired using a flow cytometer (Beckman Coulter, Miami, FL, USA), and analyzed with Multicycle software (Version 2.0, Phoenix Flow Systems, San Diego, CA, USA), with 10,000 events analyzed in each sample. The cell proliferation index (PI) was calculated as follows: PI = (S + G2/M)/(G0/G1 + S + G2/M) ×100% (25).

### Boyden chamber assay of HUVEC migration

Following siRNA and VEGF-A treatment, the HUVECs in the experimental and control groups were harvested, suspended with ECM and seeded into six-well plates at a density of 1×10^6^ cells/well. Following culture for 24 h, 2×10^5^ cells were seeded into Transwell inserts (Corning Costar, Costar, NY, USA). Inserts containing the HUVECs were placed into a six-well plate containing endothelial complete medium supplemented with 20 ng/ml VEGF-A in the lower well of a Boyden chamber. The cells were cultured for 12 to 18 h and the upper surface of the insert was then swabbed to remove unmigrated cells. The inserts were fixed with 4% paraformaldehyde for 30 min and stained with crystal violet for 20 min. Cell migration was quantified by counting the number of migrated cells, expressed as the percentage of migrated cells in the control.

### Tube formation in vitro

At 48 h after siRNA treatment, the HUVECs (4×10^4^ cells/well) in the experimental and control groups were resuspended in endothelial complete medium without serum and seeded onto 96-well plates coated with Matrigel (70 μl). Photomicrographs of the center of each well were obtained following incubation of the cells at 37°C for 48 h. The tubes were stained using a Cellomics Cytoskeletal Rearrangement kit and were analyzed with Cellomics ArrayScan (Cellomics, Pittsburgh, PA, USA). Cell images were acquired with the ArrayScan^®^ HCS Reader (Cellomics, Pittsburgh, PA, USA). Tube formation was assessed by measuring the tube length by using the Image-Pro Plus 6.0 image processing system (Media Cybernetics, Inc., Rockville, MD, USA). Data are expressed as mm/mm^2^.

### Statistical analysis

Analyses were performed using SPSS 13.0 (SPSS, Inc., Chicago, IL, USA). All values are expressed as the mean ± standard deviation. One-way analysis of variance was performed to determine the statistical significance. P<0.05 was considered to indicate a statistically significant difference.

## Results

### Von Willebrand factor expression by HUVECs

**Figure 1:**
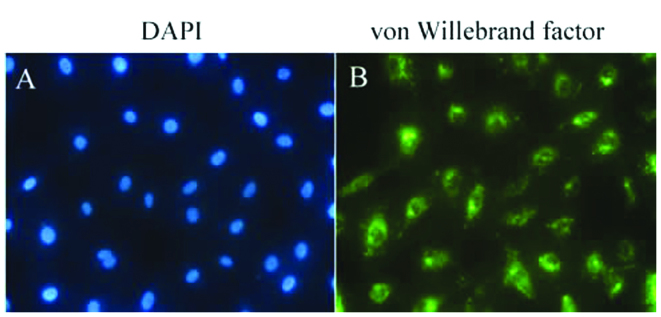
Immunofluorescence staining of HUVECs for von Willebrand factor. (A) HUVECs stained with DAPI. (B) Immunofluorescent staining of HUVECs with antibodies against von Willebrand factor. The cells exhibited a cobblestone-like appearance and were homogeneous for green fluorescence staining in the cytoplasm. HUVECs, human umbilical vein endothelial cells. Magnification ×400.

The von Willebrand factor is a glycoprotein synthesized by ECs and is commonly used as a marker for HUVECs (26). In the present study, the HUVECs exhibited a round or spindle-shaped morphology under a bright-field microscope. Under a fluorescence microscope, the majority of the HUVECs stained positive for von Willebrand factor. The cells had a cobblestone-like appearance and were homogeneous with green fluorescence in the cytoplasm (Fig. 1). No fluorescence was detected in the control cells.

### VEGF-A upregulates the expression of phosphorylated paxillin in HUVECs

**Figure 2:**
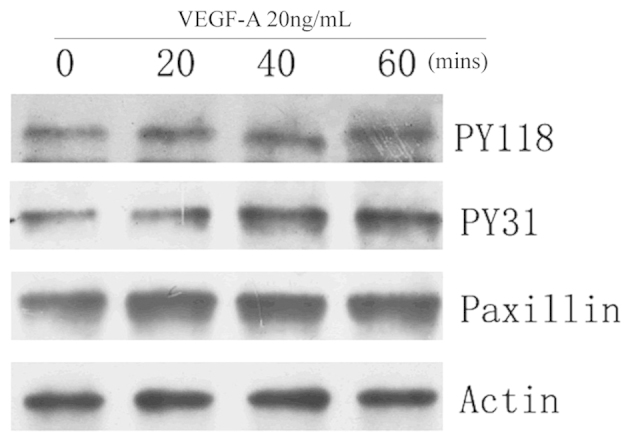
VEGF-A induces the phosphorylation of paxillin in HUVECs. The HUVECs were treated with 20 ng/ml VEGF-A for 0, 20, 40 and 60 min. Western blot analysis was performed to assess the phosphorylation of paxillin phosphorylated at PY118 and PY31 as well as total paxillin protein. Actin was used as a loading control. VEGF-A, vascular endothelial growth factor A; HUVECs, human umbilical vein endothelial cells. PY118, tyrosine 118; PY31, tyrosine31.

The present study subsequently investigated the effects of VEGF-A on the phosphorylation of paxillin in HUVECs by immunoprecipitation. The HUVECs were treated with 20 ng/ml VEGF-A for 0, 20, 40 and 60 min. Immunoblots using antibodies specific to paxillin phosphorylated at PY118 or PY31 revealed that the phosphorylation of paxillin at PY118 and PY31 increased in an incubation time-dependent manner (Fig. 2).

### siRNA-mediated knockdown decreases the mRNA and protein expression levels of paxillin in HUVECs

**Figure 3:**
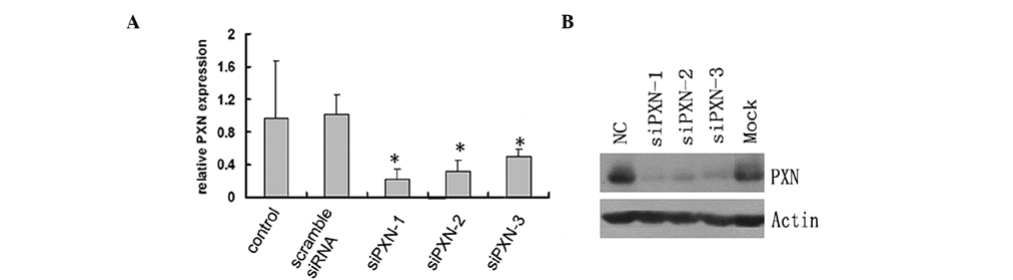
siRNA targeting PXN efficiently knocks down the expression of paxillin in HUVECs. (A) Reverse transcription quantitative polymerase chain reaction analysis of the relative mRNA expression of PXN in HUVECs, which were either mock-transfected, transfected with scramble siRNA (NC) or transfected with three individual pairs of siRNA constructs targeting PXN (siPXN-1, siPXN-2 and siPXN-3), respectively. The expression of actin was used as an internal control. Three independent experiments were performed. ^*^P<0.05, vs. control and scramble siRNA. (B) Representative western blot of the expression of PXN in the HUVECs transfected with the identical siRNA constructs. PXN, paxillin; siRNA, small interfering RNA; HUVECs, human umbilical vein endothelial cells; NC, negative control. Data are presented as the mean ± standard deviation.

The role of paxillin in the HUVECs was examined by knocking down the protein expression of paxillin by siRNA and investigating the mRNA expression by RT-qPCR. Three pairs of siRNA constructs, siPXN-1, siPXN-2 and siPXN-3, were assessed. The control scramble siRNA did not alter the relative mRNA expression of paxillin in the HUVECs; however, siPXN-1, siPXN-2 and siPXN-3 significantly decreased the mRNA expression of paxillin in the HUVECs (Fig. 3A). Consistent with the mRNA expression results, the protein expression of paxillin in the HUVECS was also significantly inhibited by the three siRNAs (Fig. 3B). The siPXN-1 construct demonstrated the most marked inhibition of paxillin among the three siRNAs (Fig. 3). Therefore, siPXN-1 was selected for use in the subsequent experiments.

### Knockdown of paxillin inhibits the VEGF-A-induced adhesion of HUVECs

**Figure 4:**
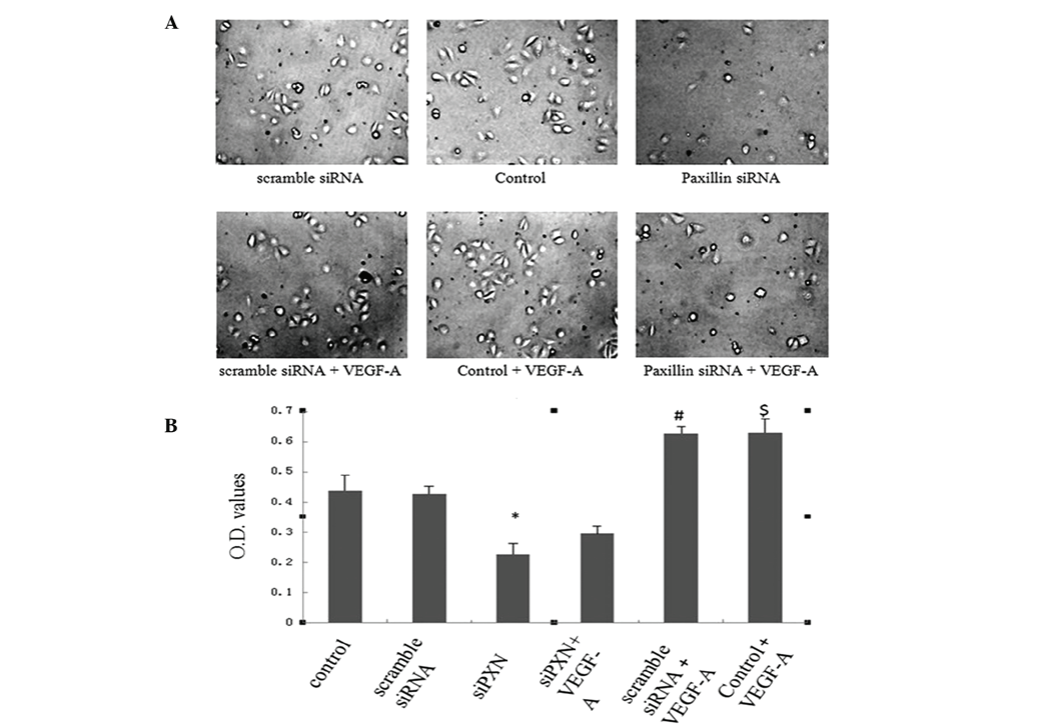
Knockdown of paxillin inhibits the VEGF-A-induced adhesion of HUVECs. The HUVECs were seeded into 96-well plates following either mock-transfection or transfection with scramble siRNA or siPXN-1, respectively. VEGF-A (20 ng/ml) was added to the medium. (A) Representative photomicrographs of the HUVECs following siRNA and VEGF-A transfection. Magnification ×200. (B) OD at a wavelength of 490 nm of the HUVECs stained with MTT. Three independent experiments were performed. ^*^P<0.05, vs. control and scramble siRNA; ^#^P<0.05, vs. control; ^$^P<0.05, vs. scramble siRNA. VEGF-A, vascular endothelial growth factor A; HUVECs, human umbilical vein endothelial cells; siRNA, small interfering RNA; OD, optical density. Data are presented as the mean ± standard deviation.

The expression of paxillin was then knocked down to assess the role of the protein in the VEGF-A-induced adhesion of HUVECs. Scramble siRNA did not alter the adhesive properties of HUVECs compared with those of the control cells. Knockdown of paxillin with siRNA significantly inhibited the adhesion of HUVECs compared with that of the control and scramble siRNA cells (Fig. 4A and B). VEGF-A treatment significantly increased adhesion of the HUVECs in the control and scramble siRNA groups; however, knockdown of paxillin with siRNA inhibited the VEGF-A-induced adhesion of HUVECs (Fig. 4A and B).

### Knockdown of paxillin inhibits the VEGF-A-induced proliferation of HUVECs

**Figure 5:**
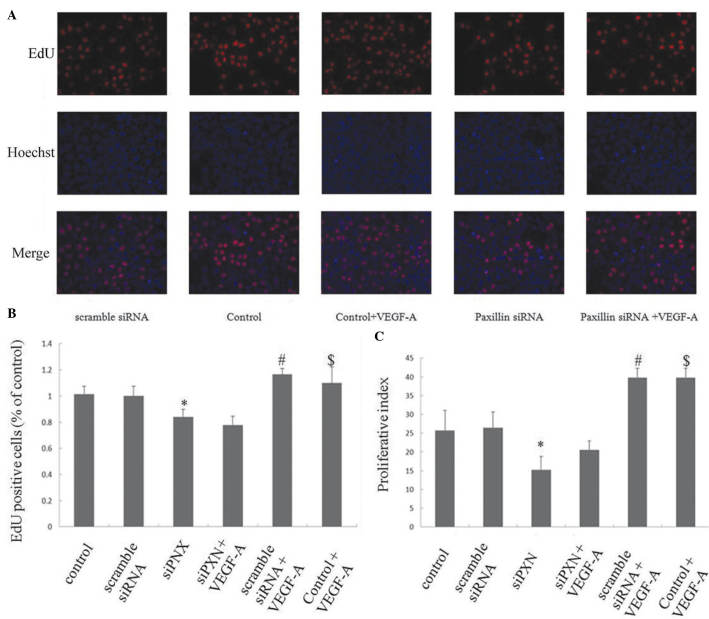
Knock down of paxillin inhibits the VEGF-A-induced proliferation of HUVECs. The HUVECs were cultured on plates following either mock-transfected or transfected with scramble siRNA or siPXN-1, respectively. VEGF-A (20 ng/ml) was added to the medium. (A) Representative photomicrographs of the HUVECs stained with EdU (red) and Hoechst 33342 (blue). Magnification ×200. (B) Percentage of EdU-positive cells. ^*^P<0.05, vs. control and scramble siRNA; ^#^P<0.05, vs. control; ^$^P<0.05, vs. scramble siRNA. (C) Cell proliferation index (PI) determined with cell cycle analysis by flow cytometry. PI = (S + G2/M)/(G0/G1 + S + G2/M) × 100%. Three independent experiments were performed. ^*^P<0.05, vs. control and scramble siRNA; ^#^P<0.05, vs. control; ^$^P<0.05, vs. scramble siRNA. VEGF-A, vascular endothelial growth factor A; HUVECs, human umbilical vein endothelial cells; siRNA, small interfering RNA. Data are presented as the mean ± standard deviation.

The effects of paxillin on the VEGF-A-induced proliferation of HUVECs were investigated using an EdU assay and cell cycle analysis by flow cytometry. Scramble siRNA did not alter HUVEC proliferation compared with that of the control; however, the knockdown of paxillin with siRNA inhibited the proliferation of the HUVECs compared with that of the control cells and cells treated with scramble siRNA (P<0.05; Fig. 5A–C). VEGF-A treatment significantly increased the proliferation of the HUVECs in the control and scramble siRNA groups; however, knockdown of paxillin inhibited the VEGF-A-induced proliferation of the HUVECs (Fig. 5A–C).

### Knockdown of paxillin inhibits the VEGF-A-induced migration of HUVECs

**Figure 6:**
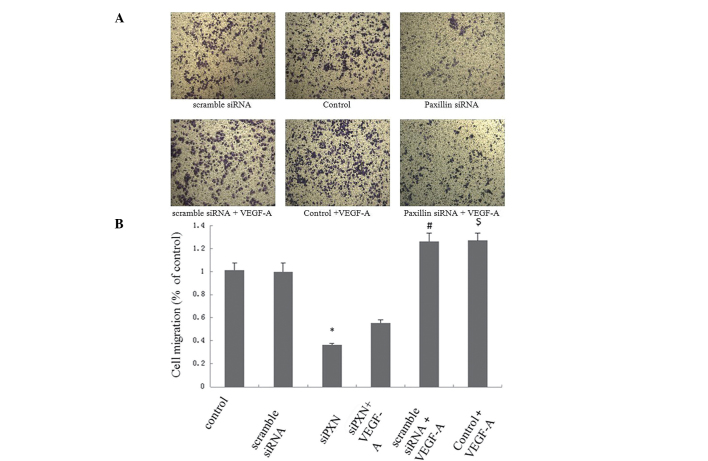
Knockdown of paxillin inhibits the VEGF-A-induced migration of HUVECs. Cells were cultured on plates following either mock-transfection or transfection with scramble siRNA or siPXN-1, respectively. HUVECs (2×10^5^ cells) were harvested and seeded into Transwell inserts. VEGF-A (20 ng/ml) was added to the lower well of a Boyden chamber. (A) Representative photomicrographs of the HUVECs in the lower well of the Boyden chamber stained with crystal violet. Magnification ×100. (B) Cell migration of the HUVECs. Cell migration was quantified by counting the number of migrated cells and expressed as a percentage of the cell migration in the control. Three independent experiments were performed. ^*^P<0.05, vs. control and scramble siRNA; ^#^P<0.05, vs. control; ^$^P<0.05, vs. scramble siRNA. VEGF-A, vascular endothelial growth factor A; HUVECs, human umbilical vein endothelial cells; siRNA, small interfering RNA. Data are presented as the mean ± standard deviation.

HUVEC migration was analyzed in a Boyden chamber using VEGF-A as a chemoattractant. The siRNA-mediated knockdown of paxillin was used to investigate the role of paxillin in the VEGF-A-induced migration of the HUVECs. No significant differences were observed in the number of migrating cells between the control cells and the cells treated with scramble siRNA; however, knockdown of paxillin significantly decreased the cell migration (P<0.01; Fig. 6A and B). VEGF-A treatment increased the number of migrated cells in the control and scramble siRNA groups (Fig. 6A and B). Of note, scramble siRNA did not affect VEGF-A-induced cell migration compared with the control; however, the knockdown of paxillin by siRNA inhibited the VEGF-A-induced migration of the HUVECs (Fig. 6A and B).

### Knock down of paxillin inhibits the VEGF-A-induced tube formation of HUVECs

**Figure 7:**
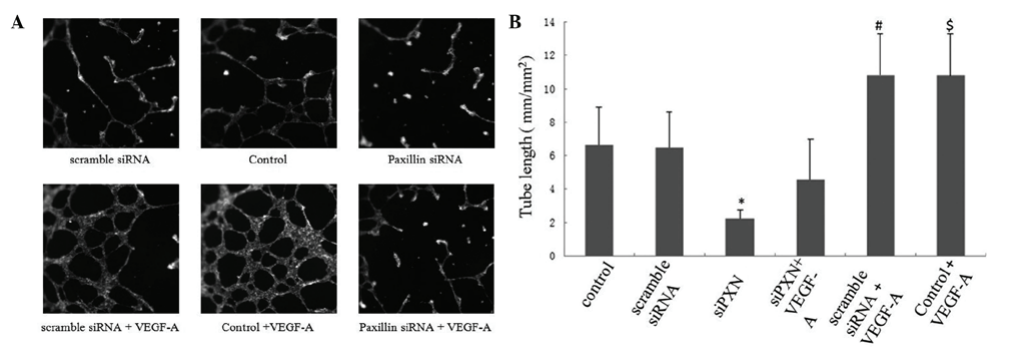
Tube formation of HUVECs is inhibited by siRNA-mediated paxillin knockdown. The HUVECs were seeded onto Matrigel 48 h after being either mock-transfected or transfected with scramble siRNA or siPXN-1, respectively. VEGF-A (20 ng/ml) was added to the medium. (A) Photomicrographs of the tubes; (B) Tube length was calculated using the Image-Pro Plus image processing system. Three independent experiments were performed. ^*^P<0.05, vs. control and scramble siRNA; ^#^P<0.05, vs. control; ^$^P<0.05, vs. scramble siRNA. VEGF-A, vascular endothelial growth factor A; HUVECs, human umbilical vein endothelial cells; siRNA, small interfering RNA. Magnification ×250. Data are presented as the mean ± standard deviation.

The effects of paxillin on VEGF-A-induced tube formation were evaluated using an *in vitro* Matrigel assay. No significant difference was observed in the number of tubes between the control group and the scramble siRNA group. Knockdown of paxillin alone significantly decreased HUVEC tube formation (Fig. 7A and B). VEGF-A treatment increased the number of tubes that formed in the control and scramble siRNA groups (Fig. 7A and B). Scramble siRNA did not affect the VEGF-A-induced tube formation compared with that of the control; however, knockdown of paxillin with siRNA inhibited the VEGF-A-induced tube formation of the HUVECs (Fig. 7A and B).

## Discussion

Angiogenesis is a natural process that occurs during healthy growth, development and wound healing. It also contributes to numerous malignant, ischemic and inflammatory diseases (9). During angiogenesis, the ECs receive angiogenic signals from growth factors to initiate proliferation, migration and the formation of a tubular structure (9). VEGF-A and its receptors are important in the promotion of the permeability, proliferation, migration and capillary tube formation of ECs (9,11,27,28) and contribute to several pathological processes, including diabetic retinopathy, retinopathy of prematurity, age-associated macular degeneration and corneal neovascularity (1,29–31). The downstream effectors and targets of VEGF receptors that mediate the diverse biological functions of VEGF-A include PKC, PLC, PI3K and FAK (9,11). However, the key targets that mediate VEGF-A-induced angiogenesis in the ECs remain to be fully elucidated. It has been reported that VEGF-A increases the phosphorylation of FAK and its substrate paxillin in the ECs (15,16). Previous studies have demonstrated that the expression of FAK, paxillin and MMPs are associated with the angiogenic activities of the ECs (32), particularly in the neovascularization observed in corneal diseases (33,34), and our previous findings suggested that FAK mediates the TNF-α-induced increase in MMPs in herpes simplex keratitis (33). *In vivo* studies have been conducted, confirming the hypothesis of the present study with regard to corneal neovascularization. However, few studies have been performed to investigate the role of paxillin in VEGF-A-mediated angiogenesis. In the present study, the siRNA-based knockdown of paxillin was used to investigate the role of the paxillin protein in VEGF-A-induced effects on the HUVECs. The results demonstrated that VEGF-A promoted the adhesion, proliferation, migration and capillary formation of the HUVECs and that the effects of VEGF-A were inhibited by paxillin knockdown. These findings suggested that paxillin is essential for VEGF-A-mediated angiogenesis in ECs.

During angiogenesis, the adhesion of ECs to each other and to the ECM is required for the growth, differentiation and survival of the cells (35). Integrins, which are heterodimeric adhesion molecules, are important in cell-cell and cell-matrix interactions (36). FAK mediates integrin signaling to recruit cytoskeletal and signaling proteins, including paxillin, which regulates the neovascularization of ECs (37). In addition, FAK is the downstream signaling molecule of VEGF and its receptors, which promote EC adhesion (15,16,37). As a downstream signaling molecule of FAK, paxillin localizes to focal adhesions and transduces adhesion and growth factor signals to regulate cell functions (14,38). In the present study, knockdown of paxillin reduced the adhesion of HUVECs in the presence and absence of VEGF-A treatment, suggesting that paxillin may mediate integrin and VEGF-A signaling in the regulation of EC adhesion, possibly though FAK.

ECs receive cues from soluble proteins and growth factors to elongate, migrate and proliferate (9) and VEGF-A, a proangiogenic factor, induces the proliferation, migration and tube-like formation of ECs (9,27,28). Consistent with previous findings, the present study found that VEGF-A treatment enhanced the proliferation, migration and tube-like formation of the ECs. VEGF-A has also been demonstrated to activate several downstream proteins. The activation of VEGFR2 by VEGF-A results in the activation of PI3K and Akt, leading to EC survival (11) and activation of the MAPK pathway, which leads to EC proliferation (39). It also leads to the activation of FAK, which induces EC migration (9,11). In the present study, the knockdown of paxillin inhibited the VEGF-A-induced proliferation, migration and tube-like structure formation of the HUVECs, suggesting that paxillin may mediate multiple signaling pathways of VEGF-A in the regulation of EC angiogenesis.

As an adapter protein within focal adhesions, paxillin provides multiple docking sites for various signaling and cytoskeletal proteins (40). Studies investigating the effects of paxillin gene disruption have demonstrated that paxillin-null cells exhibit abnormal focal adhesions, a disrupted cytoskeleton, decreased phosphorylation of FAK, decreased MAPK activation and reduced cell migration (41). Loss of Src and paxillin contributes to EC defects, including decreased proliferation, migration and angiogenesis of the ECs (42). The tyrosine kinases FAK and Src, which are activated by adhesion and growth factors, can phosphorylate paxillin and are, therefore, important in the paxillin-mediated angiogenesis of the ECs (9,11). In agreement with these previous studies, the present study found that VEGF-A treatment increased the phosphorylation of paxillin at Tyr 118 and Tyr 31. It has been reported that the phosphorylation of paxillin at Tyr 118 by Src activates the ERK signaling pathway, which leads to increased cell survival (22). In response to integrin activation, FAK activates PI3K and Akt indirectly through Src, thereby leading to increased cell survival, elongation and migration (43,44). Therefore, paxillin and FAK may recruit and activate several signaling proteins to promote these processes in the ECs.

In conclusion, the present study demonstrated that paxillin knockdown inhibited the VEGF-A-induced proliferation, migration, adhesion and capillary formation of HUVECs, suggesting that these two factors are important in angiogenesis. Therefore, paxillin may be a potential target for antiangiogenic therapies. However, how paxillin is involved in cell migration, how it regulates focal adhesions and how it interacts with VEGF and other angiogenic factors remains to be elucidated. Therefore, further understanding of the role of paxillin in angiogenesis is required to facilitate the development of antiangiogenic therapies.

## Figures and Tables

**Table I:** Primers used for amplification of the paxillin gene, reverse transcribed from human umbilical vein endothelial cell-derived mRNA.

Primer	Sequences (5′-3′)	Length (bp)	Tm (°C)	GC (%)	Size
Primer 1	Forward, AGTGCTTTGTGTGCCGGGAA	20	57.01	55.00	195 bp
	Reverse, AGGCACAGACGAAGTGCTCG	20	57.03	60.00	
Primer 2	Forward, TCATGGCCCAGGGGAAGACA	20	56.97	60.00	141 bp
	Reverse, CGCAGACTCCTTTGGCGACT	20	57.01	60.00	
Primer 3	Forward, TTCTGAACTCGACCGCCTGC	20	57.02	60.00	176 bp
	Reverse, TCTCTTTCGTCAGGGGCCCA	20	57.02	60.00	

Tm, melting temperature; GC, guanine-cytosine content.
